# Rheological and Mechanical Analyses of Felbinac Cataplasms by Using Box–Behnken Design

**DOI:** 10.3390/pharmaceutics10030088

**Published:** 2018-07-11

**Authors:** Jie Yang, Yishen Zhu, Yongqin Diao, Caiyun Yin

**Affiliations:** College of Biotechnology and Pharmaceutical Engineering, Nanjing Tech University, Nanjing 211816, China; yangj@njtech.edu.cn (J.Y.); 652085235004@njtech.edu.cn (Y.D.); 201761100874@njtech.edu.cn (C.Y.)

**Keywords:** cataplasm, felbinac, Box–Behnken design, rheology, viscoelasticity

## Abstract

Felbinac, an active pharmaceutical ingredient (API) used clinically for the treatment of osteoarthritis, has poor solubility. Felbinac cataplasm product design was investigated using rheological and mechanical analyses. Experiments using a response surface methodology based on Box–Behnken design (BBD) incorporated three independent variables: the proportions of partially neutralized polyacrylate (NP800), dihydroxyaluminum aminoacetate (DAAA), and felbinac. Statistically significant quadratic models obtained using BBD demonstrated optimal NP-800, DAAA, and felbinac cataplasm proportions of 4.78–5.75%, 0.30–0.59%, and 0.70–0.90%, respectively. Felbinac cataplasms exhibited “gel-like” mechanical property with predominantly elastic behavior. Rheological studies correlated increasing NP-800 and DAAA concentrations with increased complex modulus (G*) values that were inversely related to peeling strength. Frequency sweep and creep tests revealed decreasing tan θ values with increasing NP-800 and DAAA concentrations. G’ and G” values were higher for higher NP-800 and DAAA levels, although G” values decreased with increasing DAAA concentration. Response surface methodology was applied to develop mathematical models. Variance analysis showed that the quadratic model effectively predicted felbinac and matrix material interactions, with two verification samples upholding model predictions. Relative errors between predicted and measured G* values were 3.28% and 1.10% and for peeling strength were 1.24% and 5.59%, respectively. In conclusion, rheological and mechanical analyses of felbinac cataplasms using BBD permits optimization of cataplasms as topical drug delivery vehicles.

## 1. Introduction

Improved cataplasm excipients were developed in Japan in the 1960s and are increasingly being used in applications containing active pharmaceutical ingredients (APIs) for transdermal drug delivery system (TDDS) [1]. A cataplasm product is usually comprised of three layers: an adhesive polymer layer of crosslinked hydrogel matrix containing an API, a backing layer consisting a piece of unwoven cloth, and an anti-adhesion layer or release liner. Multivalent metal ions act as the crosslinker in the adhesive polymer layer by chelating the hydrophilic polymers.

Cataplasms should possess certain advantageous properties in order to serve as TDDS dosage vehicles, including good biocompatibility with skin, minimal dermal irritation, high drug loading capacity, excellent air permeability, and preparation using minimal organic solvents [2]. A high moisture content is also favorable for keeping the skin hydrated and reducing skin irritation. However, some cataplasm matrices produce an acidic environment, which limits application of some TDDS APIs that exhibit poor solubility in an acidic medium.

Felbinac (biphenylacetic acid), an active metabolite of fenbufen, is a non-steroidal anti-inflammatory and analgesic API. It reduces pain and swelling from inflammation resulting from prostaglandin secretion, especially in joints and muscles [3]. However, oral administration of felbinac is contraindicated, due to its adverse side-effects affecting the gastrointestinal tract; therefore, the best administration method for felbinac is TDDS. Since felbinac is poorly water-soluble and almost insoluble in an acidic matrix, it is necessary to develop and optimize an efficient method to increase felbinac solubility in TDDS formulations.

Rheological and mechanical properties of felbinac cataplasm are relevant to drug release, bio-adhesion, and mechanical stresses on skin. However, such properties should be optimized to achieve patients’ comfort. In one study [4], rheological and mechanical characterizations of novel non-aqueous ethyl cellulose gel matrices were used for TDDS. The ethyl cellulose gel was formulated successfully, and this physically crosslinked gel matrix exhibited prominent viscoelastic behavior, yield stress, and thixotropy that were suitable mechanical characteristics for TDDS. Notably, the molecular conformation of solvent greatly influenced molecular interactions in ethyl cellulose gel matrices, and this observation could assist future cataplasm development.

Partially neutralized polyacrylates containing various acrylic acid and sodium acrylate copolymerization ratios are commonly used in cataplasm formulations, including the commercially available polyacrylate product Viscomate™. In addition, crosslinkages of these polymers are stable under acidic conditions. Rheological characterization of Viscomate™ used to prepare cataplasms demonstrated that higher concentrations of dihydroxyaluminum aminoacetate (DAAA) and Viscomate™ led to increases in the elastic modulus (G’) of cataplasms, with reverse effects observed on the viscous modulus (G”) [1].

Preliminary work indicated that felbinac was almost insoluble in hydrogel matrix when pH was not controlled, which would greatly influence the drug uniformity and the formation of the cataplasm. The addition of felbinac into cataplasm can also lead to modifications in the hydrogel properties. Meanwhile, characteristics of the cataplasm have been shown to be greatly influenced by the matrix materials [1]. Since desirable attributes of felbinac cataplasm depend on optimized rheological and mechanical properties, this study investigated the effects of felbinac composition on cataplasm characteristics at certain pH using rheological and mechanical evaluations.

## 2. Materials and Methods

### 2.1. Materials and Instruments

Felbinac was bought from Energy Chemical, Ltd. (Shanghai, China). Partially neutralized polyacrylate, Viscomate™ NP-800 (NP-800), was obtained from Showa Denko K.K. (Kawasaki, Japan). Glycerin, tartaric acid, DAAA, carboxymethylcellulose sodium, diisopropanolamine and sorbitol were obtained from Sinopharm Chemical Reagent Co., Ltd. (Shanghai, China). Purified water was prepared using a Direct-Q Ultrapure Water Purification System (Millipore Corp., Billerica, MA, USA).

Physica MCR 301 Rheometer (Anton Paar, Graz, Austria) was used to evaluate the rheological behavior of the formulations. PT-501-D peeling strength tester (Precise Test Equipment, Ltd., Dongguan, China) was used to evaluate the peeling strength of the formulations. Design-Expect^®®^ software 8.06 (Stat-Ease Inc., Minneapolis, MN, USA) was used to develop and optimize the formulation.

### 2.2. Methods

#### 2.2.1. Box–Behnken Design (BBD)

Using preliminary work and literature reports, three parameters were identified that could potentially impact the quality of formulated felbinac cataplasm. The independent variables were percentages of NP-800 (X1), DAAA (X2), and felbinac (X3). Among these variables, NP-800 and DAAA as the core components of formulation in the hydrogel matrix have great effects on the properties of the cataplasms. Since felbinac is an insoluble and acidic API, it was also investigated as a major variable in the formulation optimization. The complex modulus (Y1) and peeling strength (Y2) of the prepared cataplasms were dependent responses. Design-Expect^®®^ software 8.06 was used to develop the formulation under BBD. The value ranges of independent variables and the dependent responses are shown in Table 1.

#### 2.2.2. Sample Preparation

Tartaric acid and felbinac were dispersed in water, and the suspension was adjusted to pH 7.5 using diisopropanolamine to dissolve the felbinac, which provided a stable structure and acceptable strength of felbinac cataplasms. Separately, NP-800, DAAA, carboxymethylcellulose sodium, and sorbitol were dispersed uniformly in glycerin, and the felbinac solution was then added to the mixture while it was mechanically stirred gently. The mixture was stirred for an additional ten minutes and used to coat the backing layer, then covered with the anti-adhesion layer. The samples were then cut into smaller sized pieces and sealed in a pouch until needed for rheological and peeling tests. All prepared samples were stored at room temperature (25 °C) for at least 7 days to ensure complete swelling prior to testing.

#### 2.2.3. Rheological Measurements

Rheological behavior of the formulations were evaluated at room temperature (25 °C) unless otherwise stated, using a rheometer with 25 mm diameter stainless steel parallel plates. Stress amplitude sweep and frequency sweep tests were performed in the oscillation mode, and creep tests were performed using the creep/creep recovery mode.

##### Stress Amplitude Sweep

The samples at 25 °C were exposed to increasing stress using a constant 1 Hz frequency, from 0 to 1500 Pa [5]. G* values were plotted using a logarithmic scale. The test range included the linear viscoelastic regions (LVRs) of samples, and the effects of stress were measured using oscillation tests [6].

##### Frequency Sweep

The samples were subjected to a monitored shear stress (100 Pa) over a range of frequencies (1 to 100 rad/s) within the LVRs at room temperature (25 °C). Various parameters were used to define the rheological characteristics of the samples to determine if matrix structures corresponded to those observed for ideal cataplasms from a rheological viewpoint [6].

##### Creep Test

The experiment was carried out at 25 °C with stress intensity set to 1000 Pa and maintained for 150 s. The stress was then removed instantly, and the recovery was followed for 150 s by monitoring the strain over time.

#### 2.2.4. Peeling Test

The peeling strength was evaluated for a 180° geometry using a PT-501-D peeling strength tester. Samples (250 mm × 25 mm) were pasted onto the test board and a stretching rate of 300 mm/min was used.

#### 2.2.5. Statistical Analysis

All results were evaluated statistically using ANOVA. Values of *p* < 0.05 were considered significant in all statistical comparisons.

## 3. Results and Discussion

### 3.1. BBD Statistical Analysis

Data for the complex modulus and peeling strength determinations were statistically analyzed to identify the effects of felbinac composition on cataplasm characteristics [7]. The interaction and quadratic effects among the independent variables were also evaluated.

The results of the observed values for complex modulus (Y1) and peeling strength (Y2) are shown in Table 2. Statistical analysis for the effects of X1, X2, and X3, on Y1 and Y2, was carried out using multiple regression analyses. Estimated effects, *F* values, and *p* values for the independent variables, as well as interaction and quadratic effects on derived dependent variables, were calculated, and data are presented in Table 3. 

Equations (1) and (2) represent the fitting models of the effects of Y1 and Y2 in the ranges studied for the independent variables, respectively. It is worth noting that X3 and its interaction terms were found not to exert statistically significant effects on Y2. Therefore, only effects related to X1 and X2 are applied in Equation (2).

Y1 = 1223.33 + 950.75 X1 + 294.50 X2 + 10.25 X3 + 294.25 X1X2 + 44.75 X1X3 − 50.25 X2X3 + 221.71 X1^2^ + 85.71 X2^2^ − 87.29 X3^2^ (1)

Y2 = 2.66 − 1.29 X1 − 2.40 X2 − 0.28 X1 X2 + 1.07 X1^2^ + 1.11 X2^2^ (2)

According to Table 3, results calculated using both Equations (1) and (2) were statistically significant, with *p* < 0.05, indicating that the developed models exhibited good agreement between the responses (Y1 and Y2) and the significant variables. The values of lack of fit for both Equations (1) and (2) were more than 0.05, indicating that the proposed statistical models fit well. The ANOVA results for Y1 and Y2 provided *F* values of 50.65 and 18.78, as compared to the critical values from the cutoff points for the *F* distribution (=0.05), i.e., 3.9 and 4.1, respectively. These *F* values suggest that the derived quadratic models had significant influences on the responses [8]. *R*^2^ and the adjusted *R*^2^ values for Y1 were 98.92% and 96.96%, and for Y2 were 91.25% and 86.40%, respectively, which demonstrate the accuracy of the tests and the fitness of the results with the proposed models [9].

**Figure 1:**
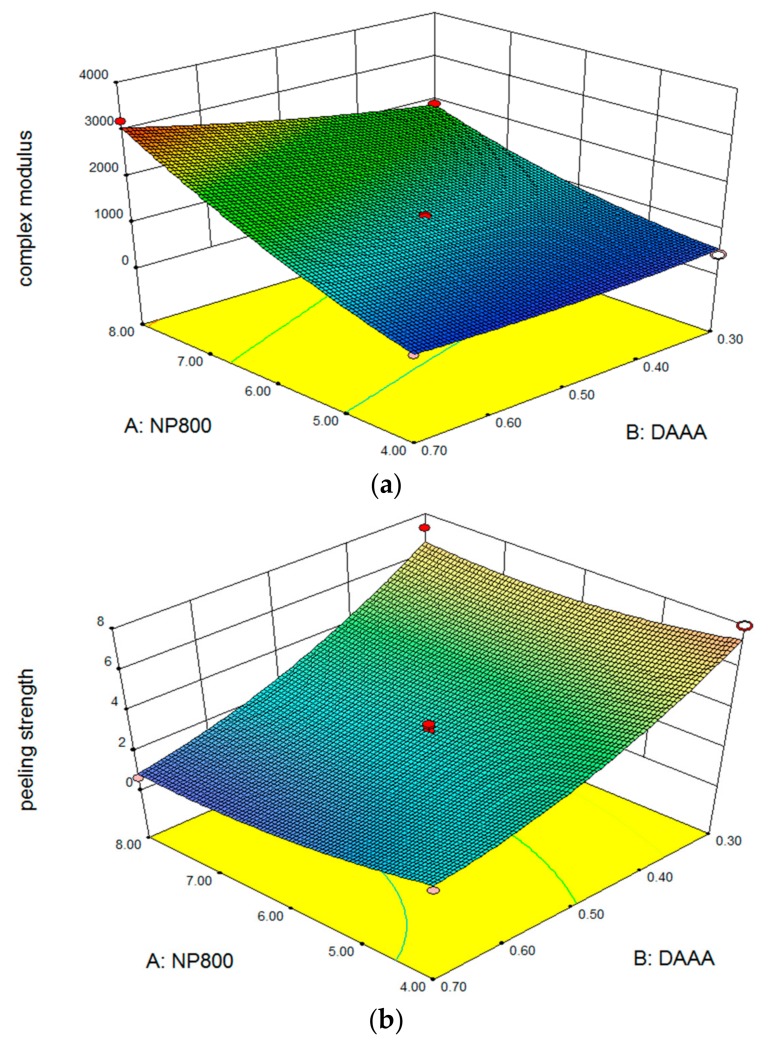
Response surface of contour plots to show effects of tested factors on complex modulus (**a**) and peeling strength (**b**).

It was concluded that the effects of X1 and X2, the quadratic effect of X1 and the interaction effect of X1X2 (Table 3) significantly influenced Y1 (*p* < 0.05). The estimated effects of X1, X2, X1^2^, and X1X2 were positive, which indicated that these independent variables had synergistic effects on Y1. According to the estimate effects, X1 was the critical independent variable in the range of the experiment, while X3, its interaction terms X1X3, X2X3, and X2^2^ were found to be insignificant. The response surface plot for the effects of X1 and X2 on Y1 were shown in Figure 1a, when X3 was set to its intermediate level (X3 = 0.8%). As the percentage of X1 and X2 increased, Y1 was increased. The *p*-value of X1X2 was 0.0077 (*p* < 0.05), indicating existence of a mutual effect exerted by X1 and X2 to increase Y1, the complex modulus. 

Y2 was significantly affected by X1, X2, X1^2^, and X2^2^, where X1, X2 had inactive or antagonistic effects on Y2 (Table 3). X1^2^ and X2^2^ have the synergistic effects. However, X1X2 was not significant. The response surface plot for the effects of X1 and X2 on Y2 (Figure 1b) indicated that increasing X1 and X2 resulted in the decrease of Y2. Furthermore, the effect of X2 on Y2 was greater than that of X1.

### 3.2. Multiple Response Optimization and Optimum Range

The combination of independent variable levels, which maximize the desirability function over the dependent responses, indicated that the optimum range for X1, X2, and X3 are 4.78–5.75%, 0.3–0.59%, and 0.70–0.90%, respectively.

Based on the optimum ranges of independent variables, two formulations, denoted A and B, were prepared and characterized accordingly, using the methods previously described. The values of Y1 and Y2 measured for formulations A and B were 925.43 Pa, 873.35 Pa, and 6.43 N/m, 3.04 N/m, respectively, while the predicted values from Equations (1) and (2) were 895.06 Pa, 883.08 Pa, and 6.51 N/m, 2.87 N/m, respectively (Table 4). The relative errors between the predicted values and the measured values were 3.28%, 1.10%, and 1.24%, 5.59%, respectively (Table 4), indicating that the results of two formulations were consistent with the predicted value and the relative errors of two formulations were acceptable.

### 3.3. Rheological Testing

#### 3.3.1. Stress Sweep

**Figure 2:**
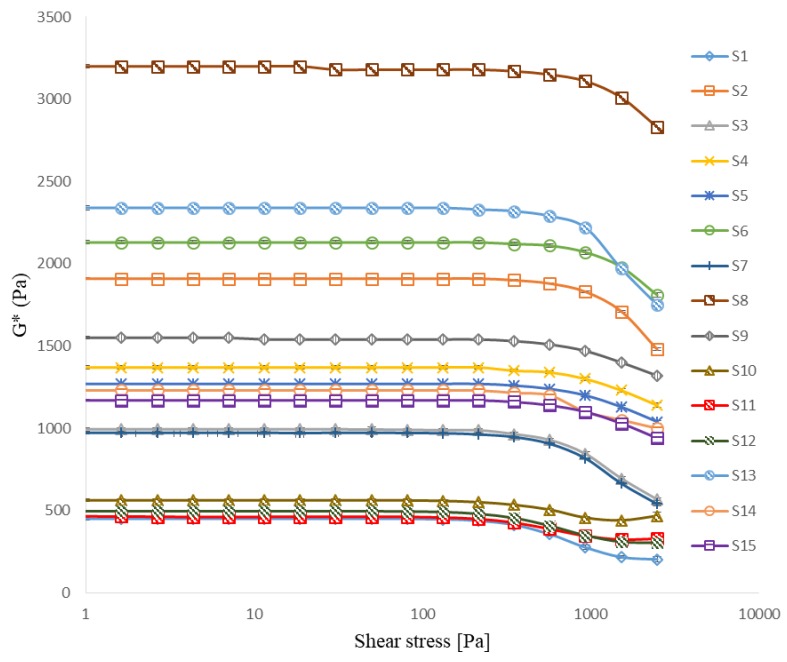
G* as a function of shear stress for felbinac cataplasm samples.

The stress sweep graphs for all prepared cataplasms are presented in Figure 2 and Figures S1–S15. In the LVRs, G* was independent at the low shear stress range. In the range studied, the upper limit of the LVRs ranged from 133 Pa to 571 Pa, and G* values ranged from 450 to 3230. The decrease in G* reflected the structural breakdown of cataplasms due to the imposition of large deformations at higher shear stress [10]. G* and critical shear stress (stress at which cataplasms began to show nonlinear viscoelastic behavior) increased with increases in NP-800 or DAAA concentration. Formulations S8 and S1 (Table 2) contained the highest and the lowest concentrations of both NP-800 and DAAA, respectively, whereby the values of G* and critical shear stress of S8 were larger than those of S1. It was expected that increased concentrations of NP-800 and DAAA would increase the density of the crosslinked matrices, as observed. This result is explained by the fact that more aluminum ions would be released at higher DAAA concentrations for a given pH, resulting in denser crosslinked matrices. However, the effect of felbinac concentration and its interaction terms on G* and critical shear stress had no statistical difference (*p* > 0.05) according to the BBD statistical analysis. Therefore, felbinac would not contribute much to the rheological and mechanical properties of cataplasms.

In general, a “liquid-like” structure of cataplasm would have an effect on the degree of drug loading, because “liquid-like” structure has lower G’ and shorter LVR, this indicated that its structure was especially breakable and could not wrap more drugs [1]. While conversely a “solid-like” structure of cataplasm would influence drug release, because the lower G’ values of matrix cause the faster drug release rate [11]. Cataplasm with stronger matrix structure would retain the drug diffusion through the matrix. G* values between 800 to 1000 Pa are in the ideal range for cataplasms with suitable viscoelastic properties [12]. Therefore, the G* values of S3 and S7 at 994 ± 6 Pa and 973 ± 8 Pa, respectively, were consistent with suitable drug loading and release characteristics for drug delivery applications.

#### 3.3.2. Oscillatory Frequency Sweep

**Figure 3:**
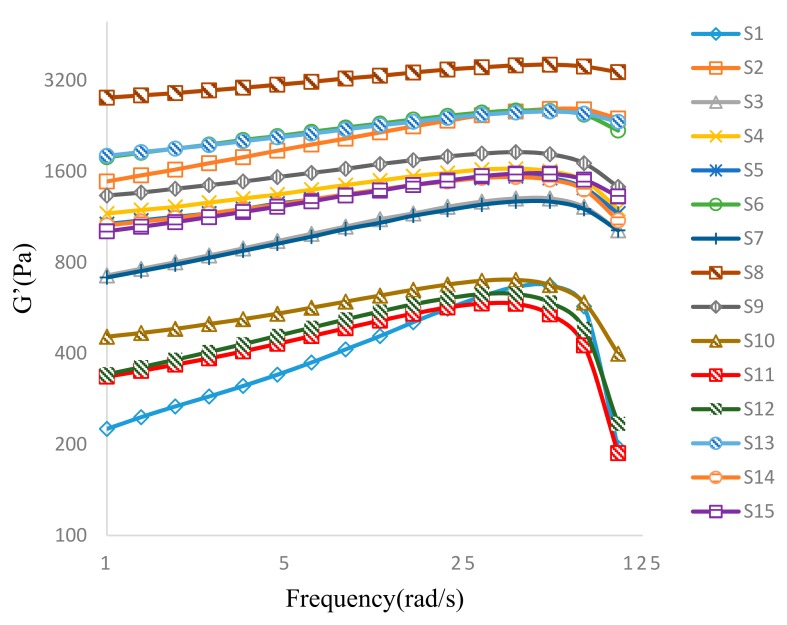
Evolution of G’ as a function of the applied frequency for samples of felbinac cataplasms.

**Figure 4:**
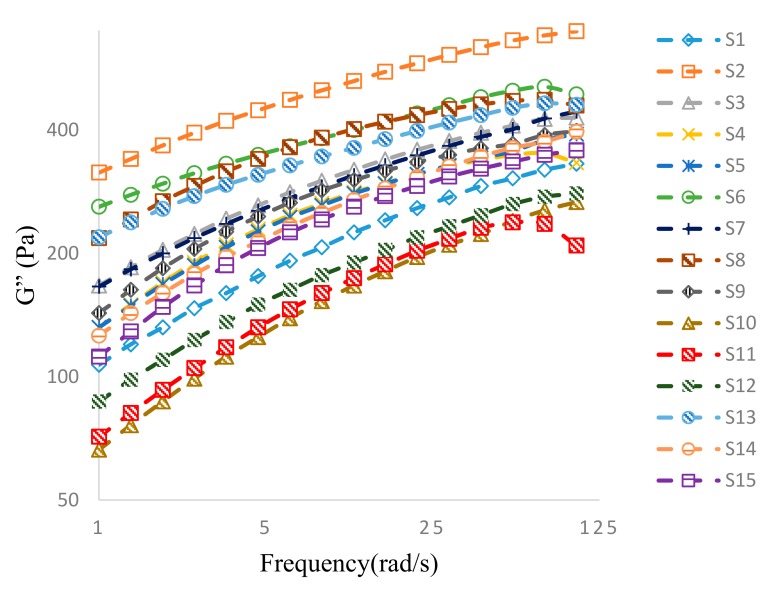
Evolution of G” as a function of the applied frequency for samples of felbinac cataplasms.

Felbinac cataplasms have previously been observed to exhibit “gel-type” mechanical property, with G’ > G’’ and tan < 1.0 in both the oscillatory stress sweep and frequency sweep, suggesting predominantly elastic behavior [13]. The plots of the variations in G’ and G” of samples studied using BBD are displayed in Figure 3 and Figure 4, respectively. At low oscillation frequencies, a predominant elastic behavior was observed, G’ > G”, and both G’ and G” values increased with increase in frequency. Frequency adjustment had a greater impact on G’ than that on G’’, as expected for a typical three-dimensional gel network [6]. When the oscillation frequency exceeded 60 rad/s, both G’ and G” exhibited downward trends for all samples tested, indicating destruction of the three-dimensional gel networks by high shear stress. From a micro perspective, three-dimensional structure would be distorted by the rotation of the parallel plate at frequencies over 60 rad/s. Subsequently, the ionic bonds for crosslinks between the aluminum ions and carboxylic groups of polymers were disrupted by the effects of potential energy. Thus, a transition from a structured gel behavior to that of concentrated polymer chains followed that generated steep decreases in G’ as a result (Figure 3). However, the disruptive behaviors seen in all tested samples occurred at very high oscillatory frequencies, beyond conditions appropriate for normal cataplasm applications. Hence, felbinac cataplasms formulated in this study possess rheological properties suitable for use [4].

Comparing the G’ and G’’ plots of S8 and S2 (Figure 3), in which the DAAA concentrations varied while other variables remained unchanged (Table 2), the G’ value of S8 was larger than that of S2, while the G’’ value of S8 was smaller. Therefore, a higher DAAA concentration in S8 decreased the G” value. Theoretically, the G” value is affected by the concentration of acrylic acid, whereby the acrylic acid in the polymer structure promotes closer contact with the skin [14]. Therefore, it could be suggested that a higher DAAA concentration decreased the G” value by steric hindrance in the matrix caused by higher crosslinking density. Consequently, due to greater steric hindrance induced by higher DAAA concentration, acrylic acid on the “outside” layer of cataplasm could not interact readily with the skin [15].

**Figure 5:**
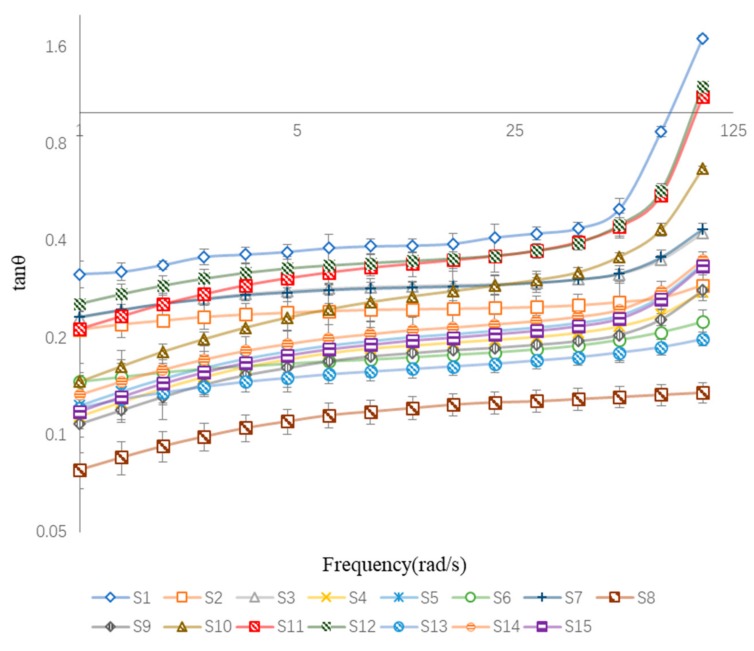
The tan θ as a function of frequency for samples of felbinac cataplasms.

The loss tangent (tan θ), which is described as the ratio of G” and G’ during an oscillatory cycle (tan θ = G″/G′), is a measure of the relative contributions of the elasticity and viscosity of a material [16]. A tan θ value close to 1 describes a “liquid-like” system, whereas a value closer to 0 describes a more “solid-like” system. Plots of tan θ for all BBD-tested samples are displayed in Figure 5. Only tan θ of S1, S11, and S12 were greater than 1 at a high frequency, while most samples exhibited predominantly “solid-like” characteristics. However, the samples containing low concentrations of both NP-800 and DAAA exhibited “liquid-like” characteristics at high frequency. These results indicate that a decrease of NP-800 and DAAA concentrations would also decrease crosslinking density in the cataplasms and are consistent with results predicted by this BBD study.

#### 3.3.3. Creep-Recovery Study

**Figure 6:**
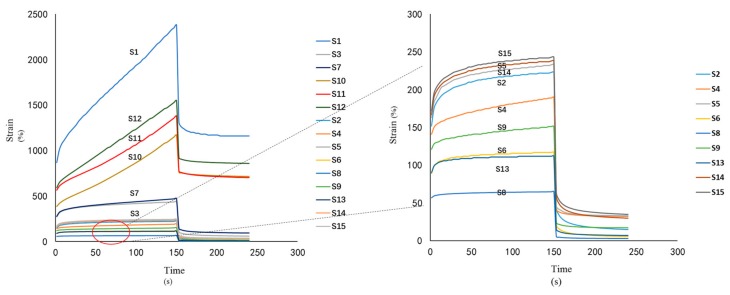
Strain–time plots at constant stress level applied to the samples tested.

Strain as a function of time for the cataplasms was studied at constant stress level (Figure 6 and Figures S16–S30. All samples showed creep behavior similar to that of a Kelvin–Voigt body [17], where strain values increased faster at the beginning of the experiment and slowed down later. After the stress was removed, the strain did not relax to a zero-stress state, but instead reached an equilibrium state after a time, thus indicating that polymers were crosslinked in all samples tested. However, the degrees of crosslinking varied due to strain differences produced by the samples. Consequently, samples with higher NP-800 and DAAA concentrations were subject to lower strains and recovered more from deformation, further demonstrating that NP-800 and DAAA played important roles in the behaviors of cataplasm formulations. Conversely, changes in felbinac concentration had little effect on the creep characteristics of cataplasms. Overall, as compared with other samples, S3 and S7 exhibited moderate deformations that would be beneficial and biocompatible for skin contact.

### 3.4. Peeling Test

In general, cataplasms are faced with the problem of insufficient viscosity, and the peeling strength values of cataplasms are relatively low in comparison to poly(acrylic acid)/laponite nanocomposite hydrogels [18] and the traditional pressure-sensitive patch [19]. Therefore, the maximum value of peeling strength is chosen as an ideal value in this study. The various cataplasm sample formulations (Table 2) exhibited significant differences in peel test results. Peeling strength increased with decreases in NP-800 and DAAA concentrations (Figure 1b and Figure S1), with lowest NP-800 and DAAA concentrations exhibiting the highest peeling strength. Meanwhile, felbinac cataplasms with higher G’ presented stronger, more solid-like structures; such cohesive cataplasms provided higher resistance to deformation and lower peeling strength. As increases in NP-800 and DAAA concentrations increased crosslinking strength and density, cataplasm fluidity would be reduced, and would negatively impact diffusion capacity into adherents, resulting in decreased peeling strength. However, any change in felbinac concentration had no obvious influence on cataplasm peeling strength, although felbinac of lower concentration exhibited even lower effects on the cataplasm. Currently, the peel strength of cataplasms can be improved by adding a thickening agent.

## 4. Conclusions

BBD was successfully implemented to evaluate the effects of concentration adjustments of NP-800, DAAA, and felbinac on complex modulus and peeling strength of felbinac cataplasms. ANOVA results revealed that the proposed regression models based on BBD were consistent with experimental data, and that the statistical models were effective. The optimal NP-800, DAAA, and felbinac levels were 4.78–5.75%, 0.30–0.59%, and 0.70–0.90%, respectively. Results using verification samples demonstrated that the optimal ranges were statistically satisfactory. Furthermore, felbinac cataplasms were demonstrated to be crosslinked structures using rheological and mechanical properties analyses. Stress sweep and oscillatory frequency sweep analyses indicated that felbinac cataplasm exhibited a “gel-type” mechanical spectrum with elastic behavior, while loss tangent analysis suggesting predominant “solid-like” characteristics were also observed. High elastic deformation observed in the creep-recovery study indicated varying degrees of felbinac cataplasm crosslinking. While high crosslinking density decreased peeling strength, felbinac concentration generally exerted no statistically significant effects on cataplasm physical properties. Based on these results, cataplasm formulations should therefore be explored further for their use as TDDS vehicles.

## Figures and Tables

**Table 1:** The levels of independent variables and the range of dependent responses of felbinac cataplasms using Box–Behnken design.

Independent Variables	Level	
	High	Low
NP800 (%)	8	4
DAAA (%)	0.7	0.3
Felbinac (%)	1	0.6
Dependent responses	Aim	
Complex modulus (Pa)	800–1000	
Peeling strength (N/m)	maximize	

**Table 2:** Experimental design and results of the felbinac cataplasms.

No.	Independent Variables			Dependent Responses *	
	X1 (%)	X2 (%)	X3 (%)	Y1 (Pa)	Y2 (N/m)
S1	4	0.3	0.8	450 (±5)	7.96 (±0.19)
S2	8	0.3	0.8	1910 (±8)	7.33 (±0.007)
S3	6	0.3	1.0	994 (±6)	5.81 (±0.55)
S4	6	0.7	1.0	1370 (±5)	2.51 (±0.002)
S5	6	0.5	0.8	1270 (±3)	2.86 (±0.19)
S6	8	0.5	0.6	2130 (±5)	2.00 (±0.08)
S7	6	0.3	0.6	973 (±8)	5.73 (±0.27)
S8	8	0.7	0.8	3200 (±7)	1.06 (±0.04)
S9	6	0.7	0.6	1550 (±5)	1.25 (±0.20)
S10	4	0.7	0.8	563 (±7)	2.82 (±0.005)
S11	4	0.5	0.6	465 (±3)	5.80 (±0.16)
S12	4	0.5	1.0	496 (±3)	5.72 (±0.34)
S13	8	0.5	1.0	2340 (±9)	1.61 (±0.001)
S14	6	0.5	0.8	1230 (±8)	2.36 (±0.14)
S15	6	0.5	0.8	1170 (±7)	2.55 (±0.3)

* Each reading for Y1 and Y2 represents an average of three measurements (*n* = 3) and SD < 5% of the mean.

**Table 3:** ANOVA for quadratic model hydrogel properties of felbinac cataplasm.

Source	Y1				Source	Y2			
	Estimated Effect	*F* Value	*p* Value	*R* ^2^		Estimated Effect	*F* Value	*p* Value	*R* ^2^
Model	1223.33	50.65	0.0020 *	0.9892	Model	2.66	18.78	0.0002 *	0.9125
X1	950.75	386.30	<0.0001 *		X1	−1.29	18.34	0.0020 *	
X2	294.50	37.07	0.0017 *		X2	−2.40	63.67	<0.0001 *	
X3	10.25	0.045	0.8406		X1X2	−0.28	0.44	0.5230	
X1X2	294.25	18.50	0.0077 *		X1^2^	1.07	5.9	0.0381 *	
X1X3	44.75	0.43	0.5419		X2^2^	1.11	6.37	0.0325 *	
X2X3	−50.25	0.54	0.4956						
X1^2^	221.71	9.70	0.0264 *						
X2^2^	85.71	1.45	0.2826						
X3^2^	−87.29	1.50	0.2748						
Source		Lack of Fit		*R* ^2^		Adj *R*^2^		Adeq Precisior	
Y1		0.0801		0.9892		0.9696		24.149	
Y2		0.0669		0.9125		0.8640		13.710	

Note: * indicates significant effect of this factor on the dependent response. Abbreviations: X1^2^, X2^2^, X3^2^ are the quadratic terms for the factors, X1X2, X1X3, X2X3 are the interaction terms between the factors.

**Table 4:** Statistical comparison of predicted and measured results for Y1 and Y2 of optimized felbinac cataplasms.

Formulation	Predicted Value	Measured Value	Relative Error	Predicted Value	Measured Value	Relative Error
	Y1 (Pa)	Y1 (Pa)	(%)	Y2 (N/m)	Y2 (N/m)	(%)
A	895.06	925.43	3.28	6.51	6.43	1.24
B	883.08	873.35	1.10	2.87	3.04	5.59

## Supplementary Materials

The following are available online at http://www.mdpi.com/1999-4923/10/3/88/s1, Figures S1–S15: G* as a function of shear stress for felbinac cataplasm sample S1–S15 with error line (*n* = 3), Figures S16–S30: Strain-time plots at constant stress level applied to the sample S1–S15 tested with error line (*n* = 3).
