# Supplementary material for: Rheological and Mechanical Analyses of Felbinac Cataplasms by Using Box–Behnken Design
Source: Pharmaceutics. 2018 Jul 11;10(3):88. doi: 10.3390/pharmaceutics10030088 (PMC6161182; doi:10.3390/pharmaceutics10030088)
Supplement: Supplementary file 1 [file pharmaceutics-10-00088-s001.pdf]

Pharmaceutics **2018**, *10*, 88, doi:10.3390/pharmaceutics10030088

Supplementary Materials: Rheological and Mechanical Analyses of Felbinac Cataplasms by Using Box–Behnken Design

Jie Yang, Yi-Shen Zhu, Yongqin Diao and Caiyun Yin

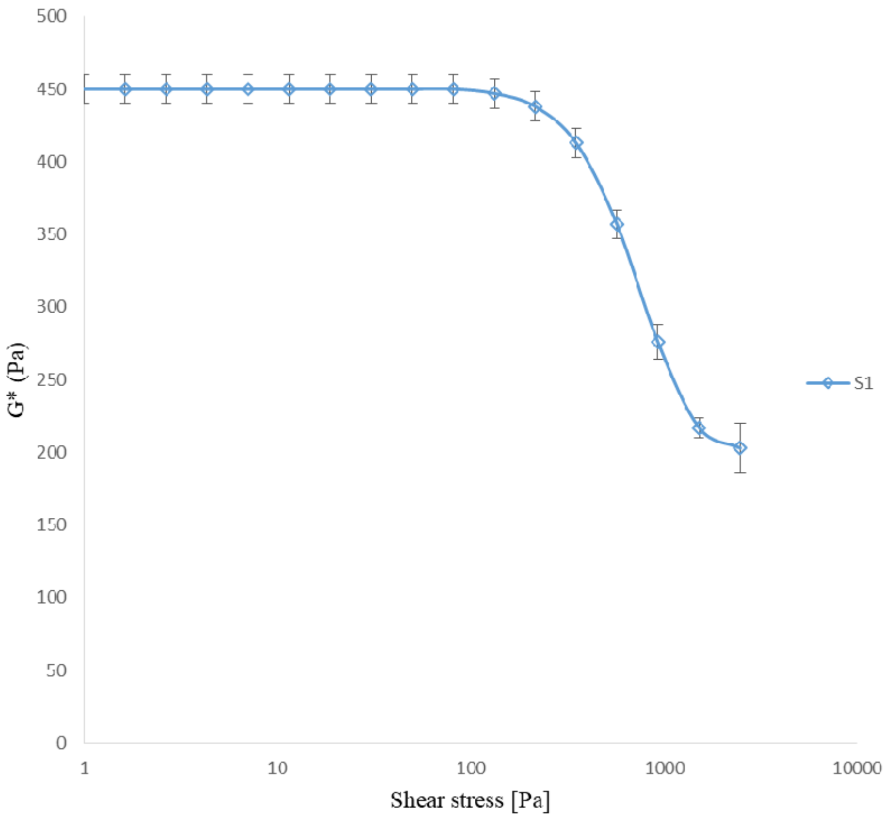

Figure S1. G* as a function of shear stress for felbinac cataplasm sample S1 with error line (n = 3).

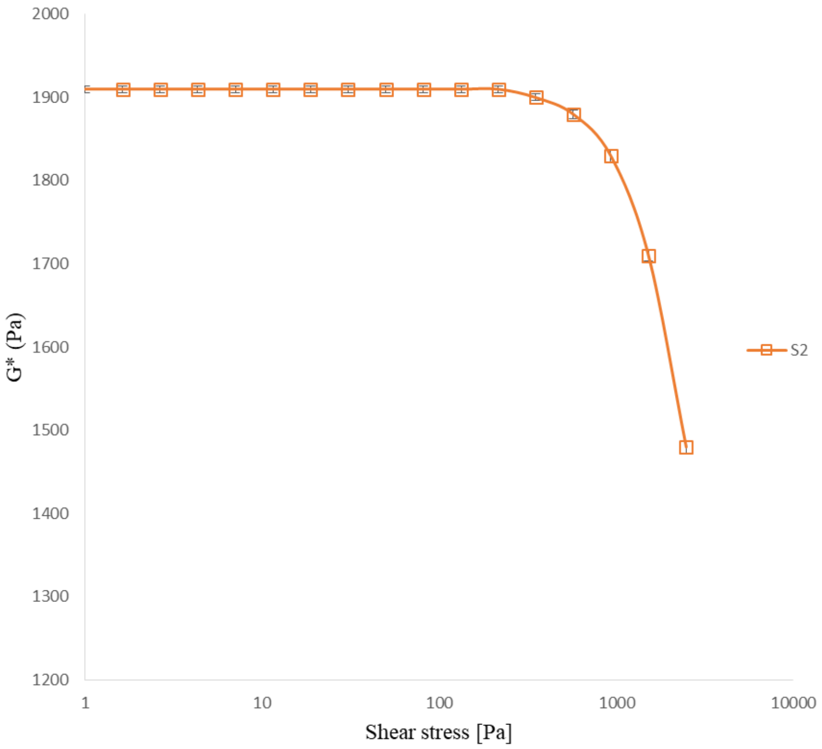

Figure S2. G* as a function of shear stress for felbinac cataplasm sample S2 with error line (*n* = 3).

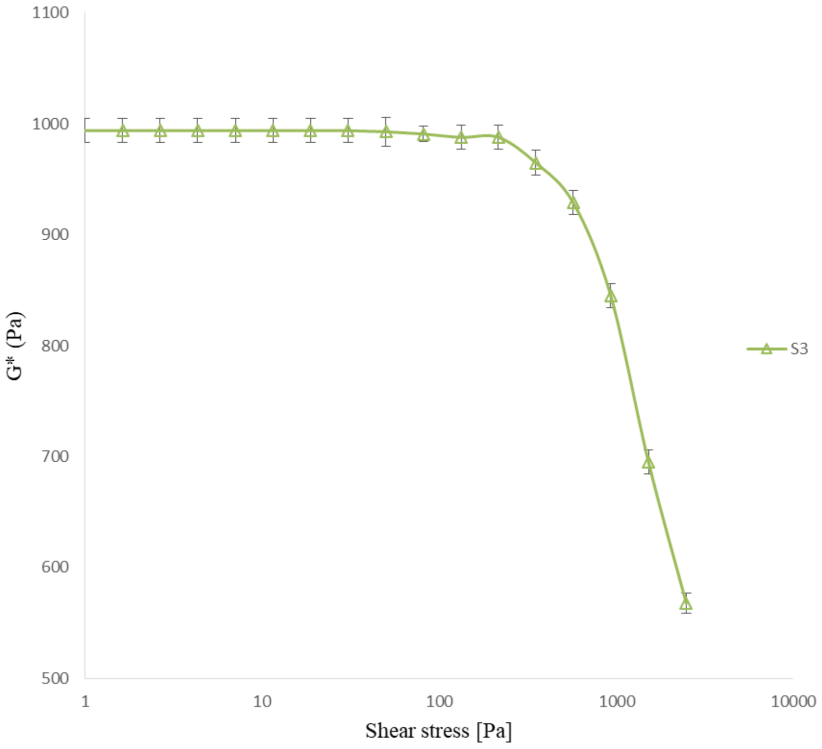

Figure S3. G* as a function of shear stress for felbinac cataplasm sample S3 with error line (*n* = 3).

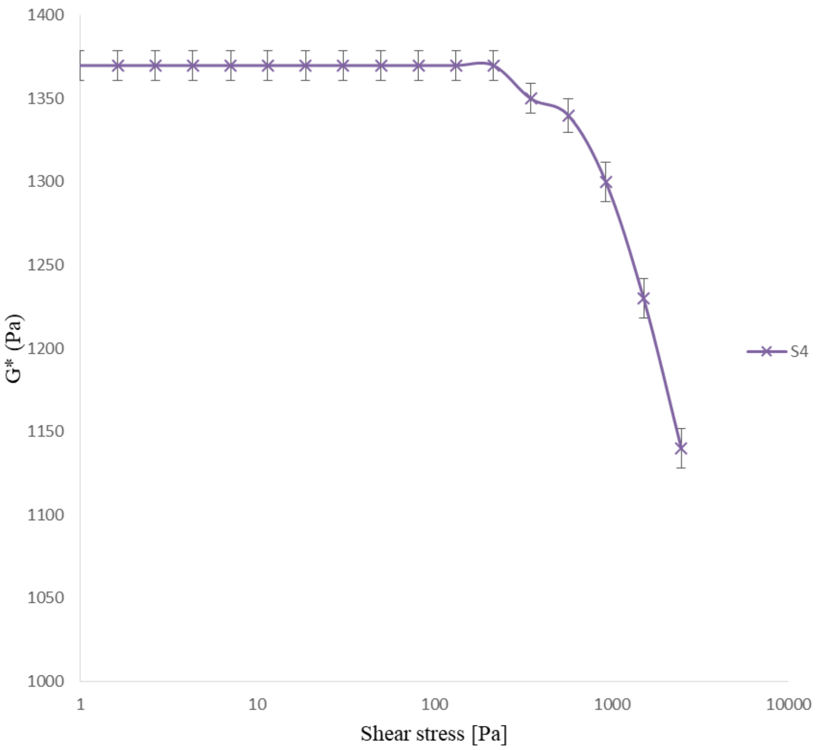

Figure S4. G* as a function of shear stress for felbinac cataplasm sample S4 with error line (n = 3).

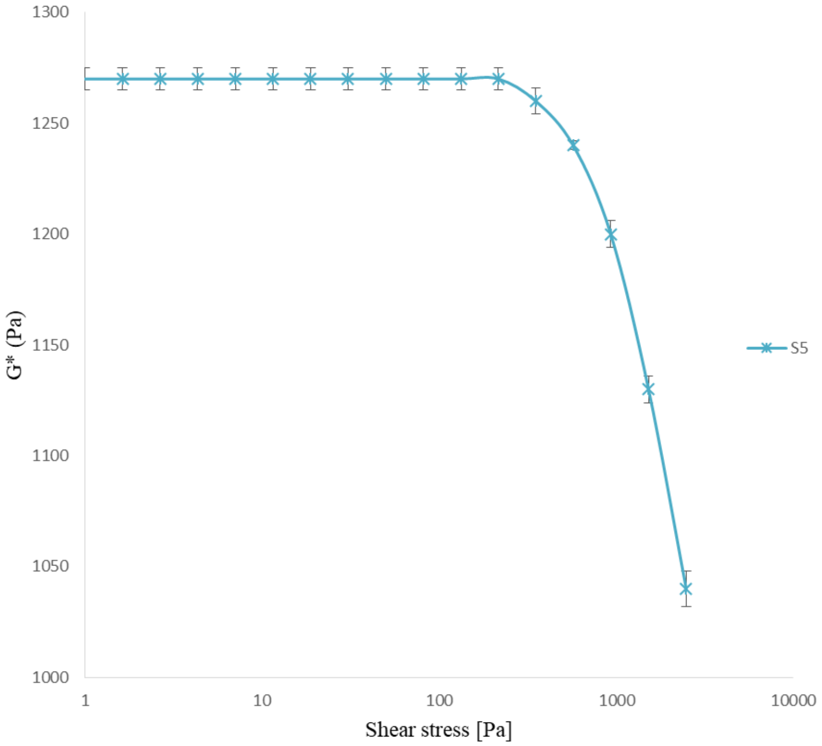

Figure S5. G* as a function of shear stress for felbinac cataplasm sample S5 with error line (n = 3).

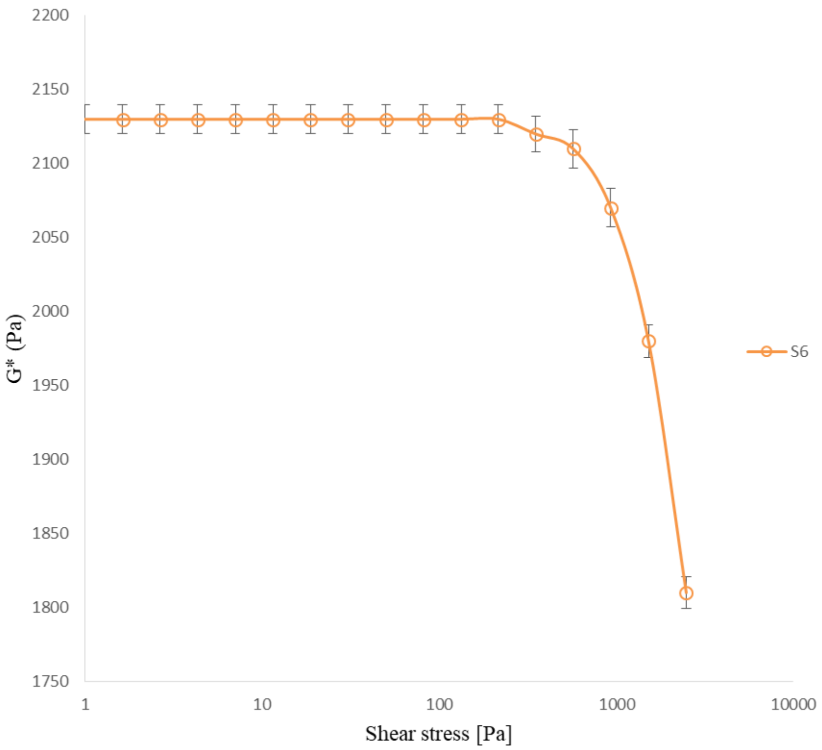

Figure S6. G* as a function of shear stress for felbinac cataplasm sample S6 with error line ($n = 3$).

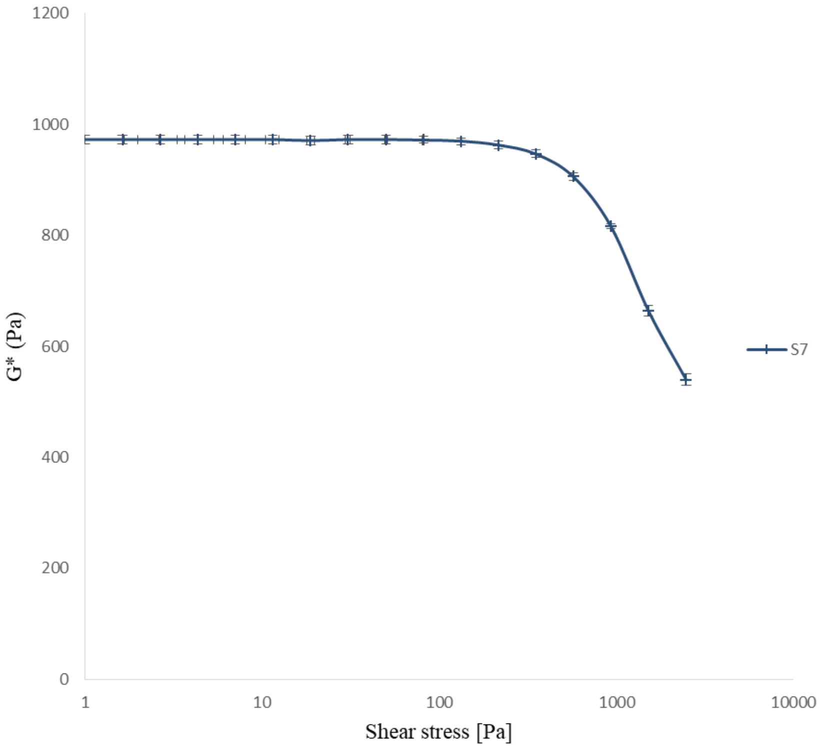

Figure S7. G* as a function of shear stress for felbinac cataplasm sample S7 with error line ($n = 3$).

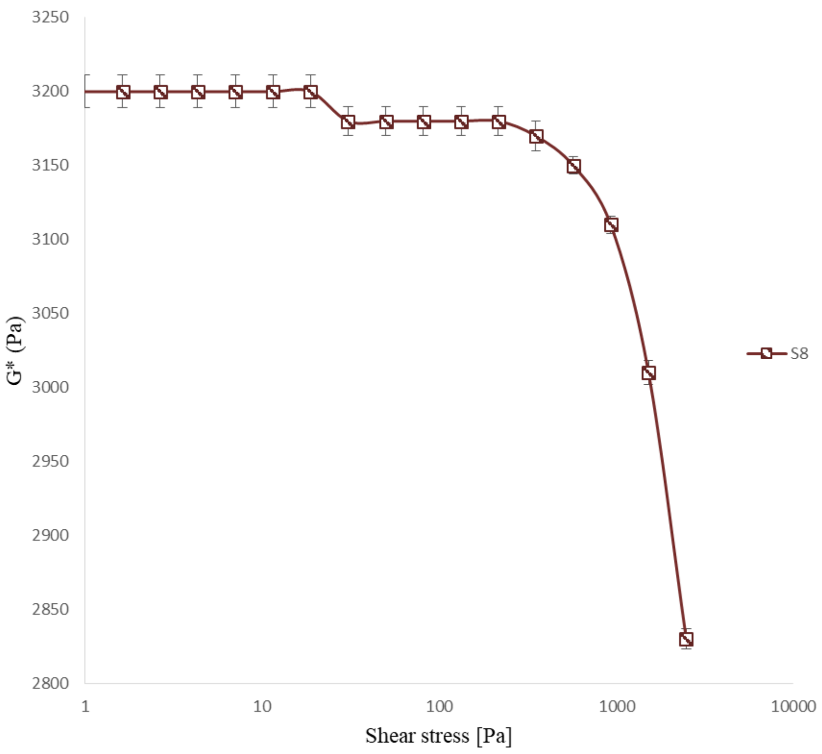

Figure S8. G* as a function of shear stress for felbinac cataplasm sample S8 with error line (*n* = 3).

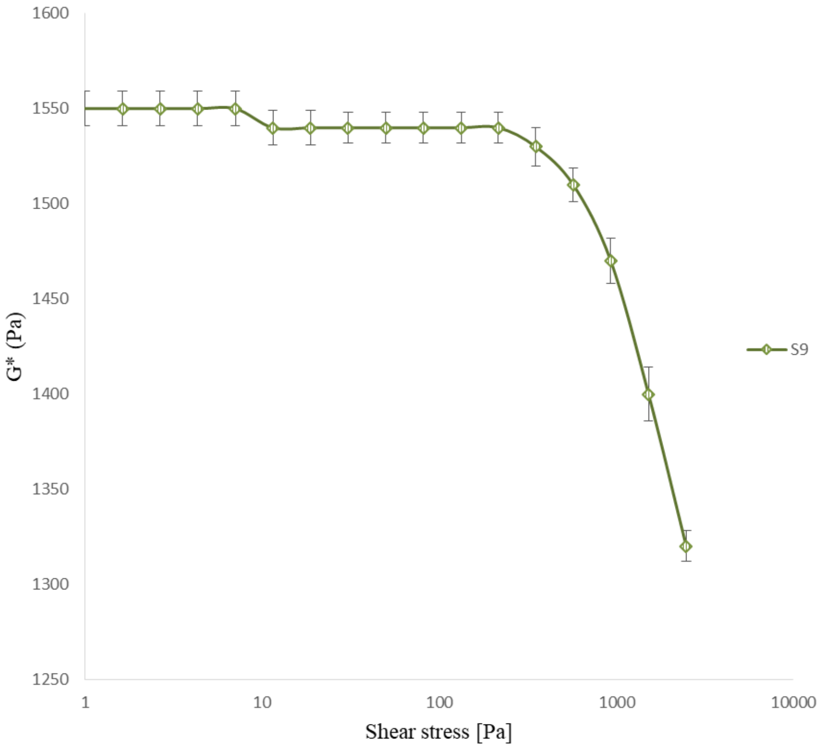

Figure S9. G* as a function of shear stress for felbinac cataplasm sample S9 with error line (*n* = 3).

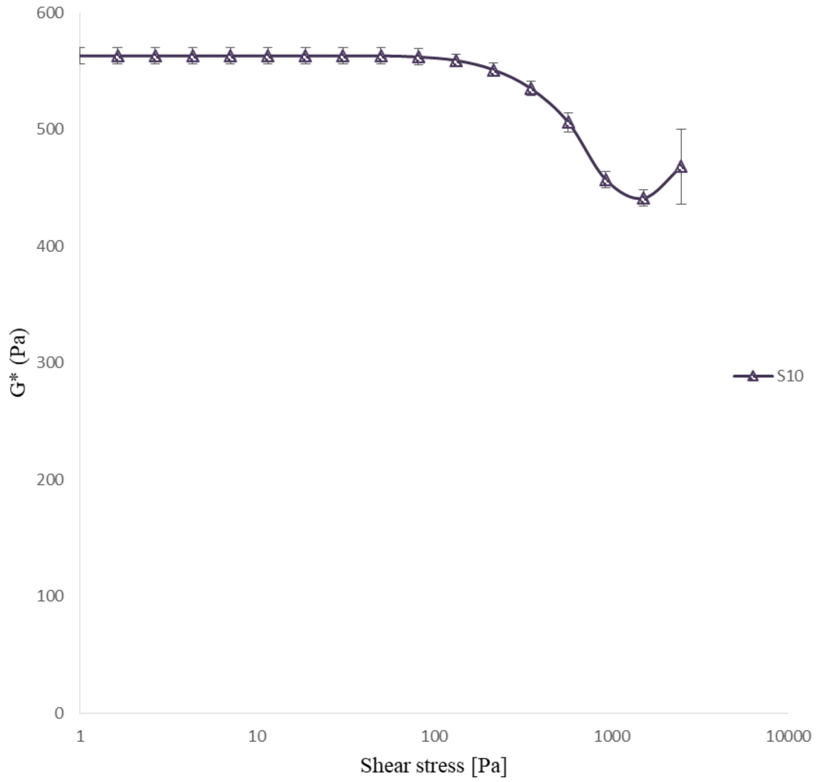

Figure S10. G* as a function of shear stress for felbinac cataplasm sample S10 with error line (n = 3).

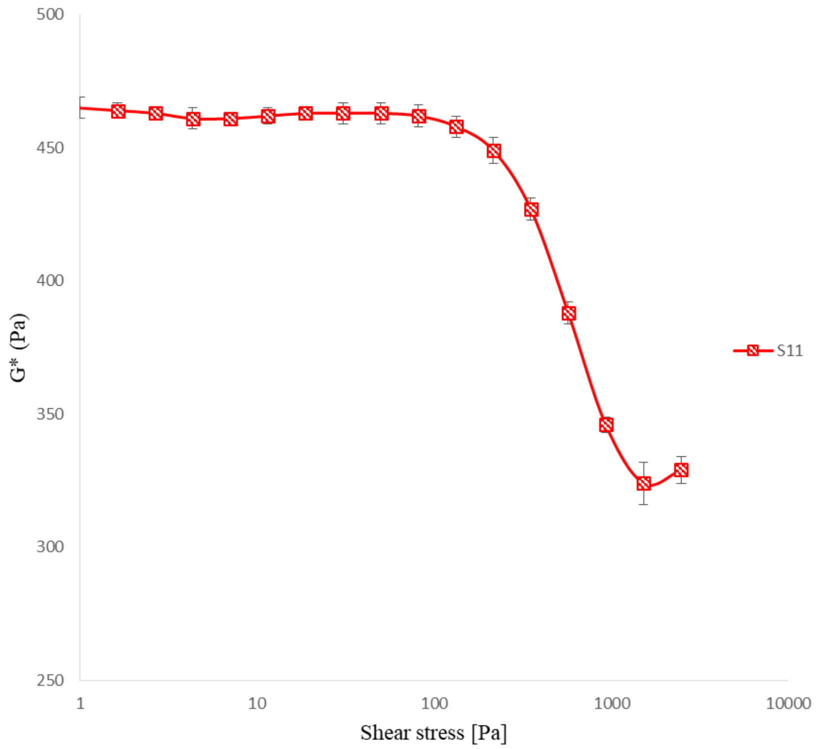

Figure S11. G* as a function of shear stress for felbinac cataplasm sample S11 with error line (n = 3).

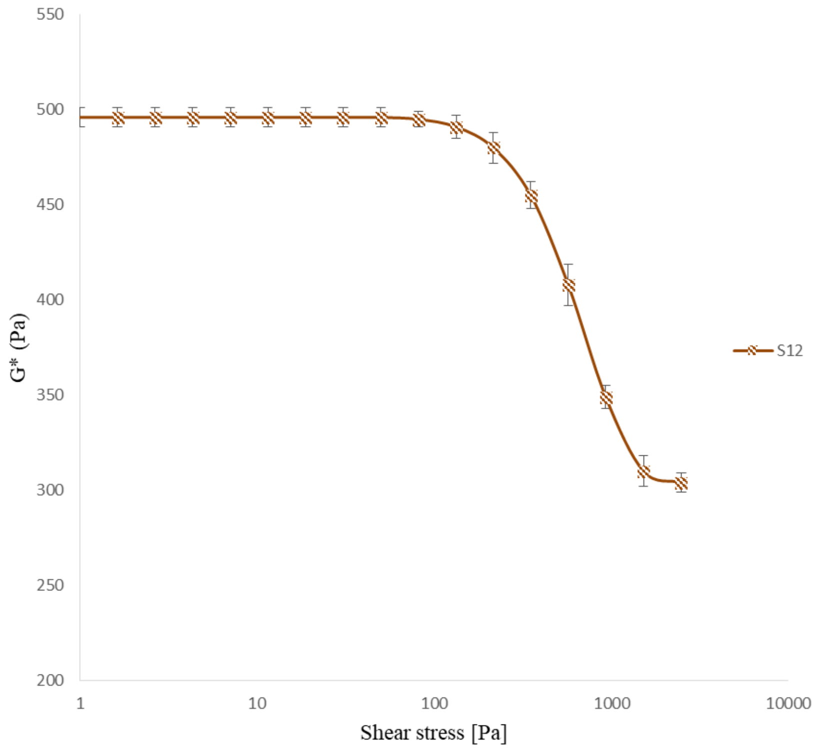

Figure S12. G* as a function of shear stress for felbinac cataplasm sample S12 with error line (*n* = 3).

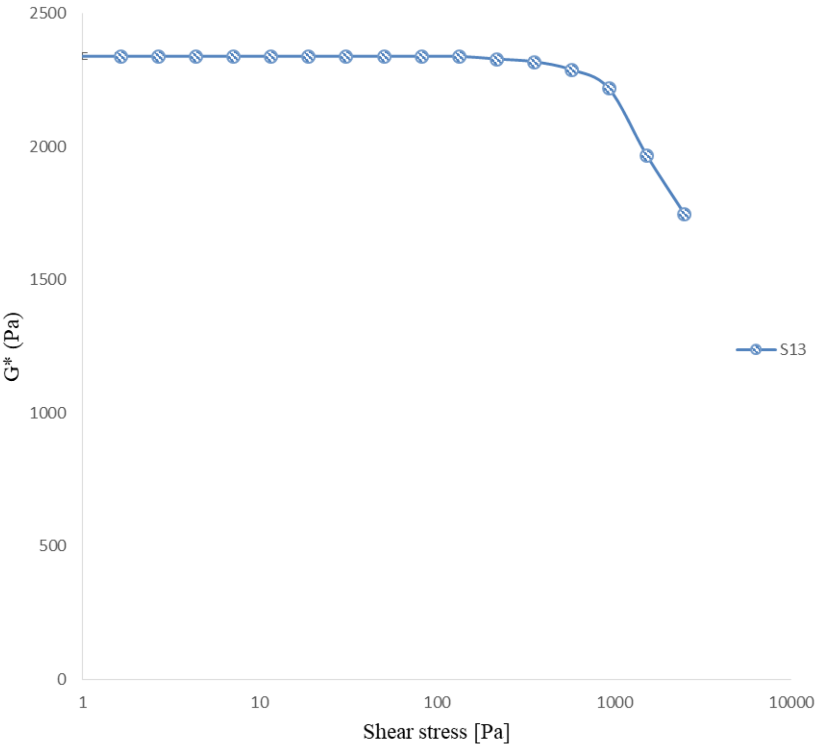

Figure S13. G* as a function of shear stress for felbinac cataplasm sample S13 with error line (*n* = 3).

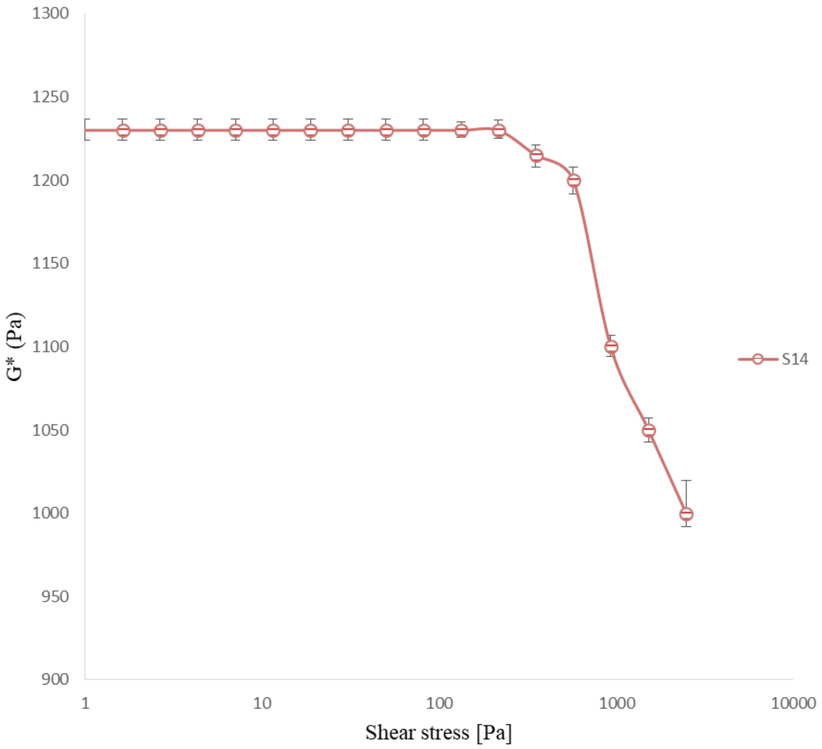

Figure S14. G* as a function of shear stress for felbinac cataplasm sample S14 with error line (*n* = 3).

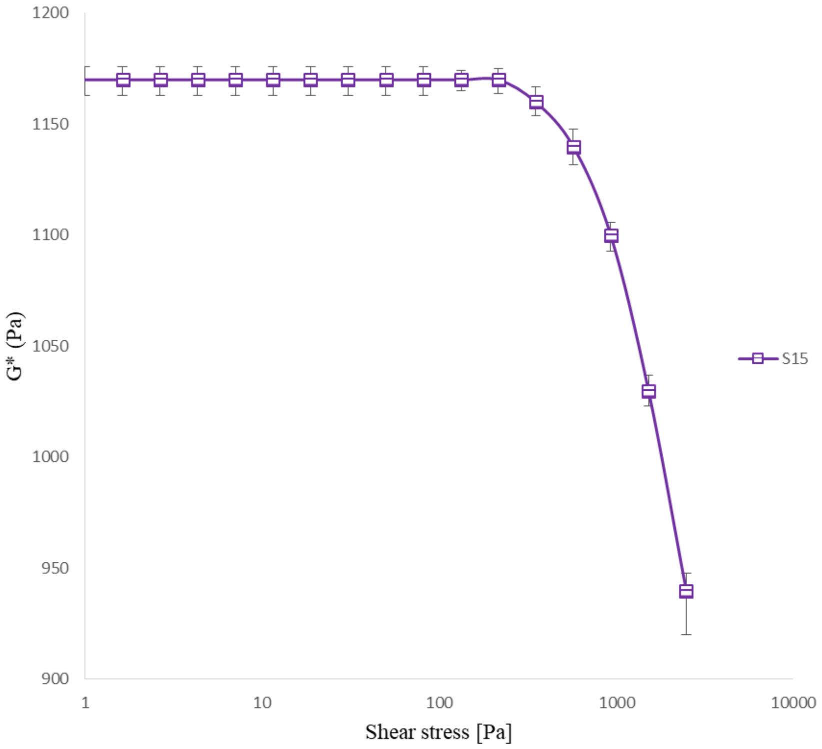

Figure S15. G* as a function of shear stress for felbinac cataplasm sample S15 with error line (*n* = 3).

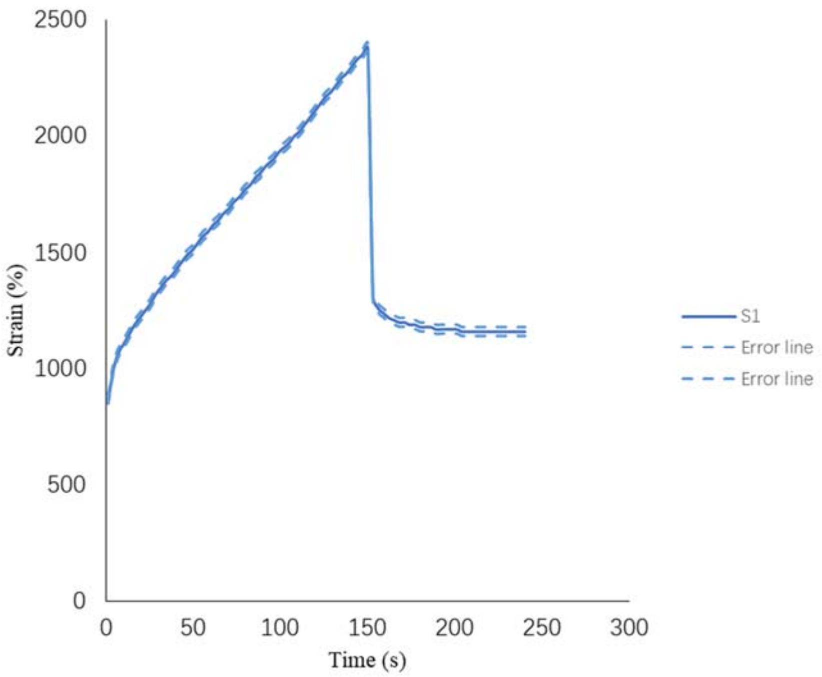

Figure S16. Strain-time plots at constant stress level applied to the sample S1 tested with error line ($n = 3$).

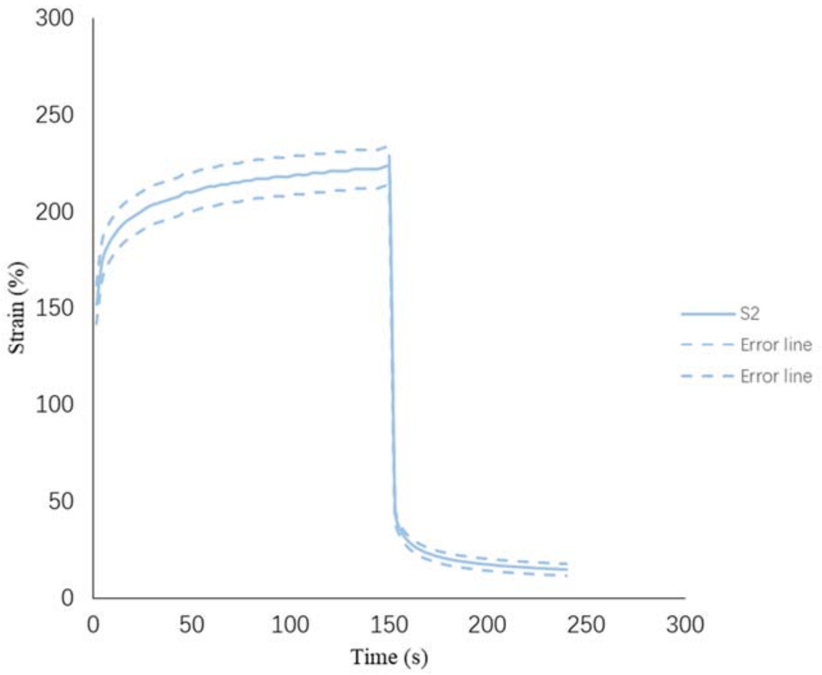

Figure S17. Strain-time plots at constant stress level applied to the sample S2 tested with error line ($n = 3$).

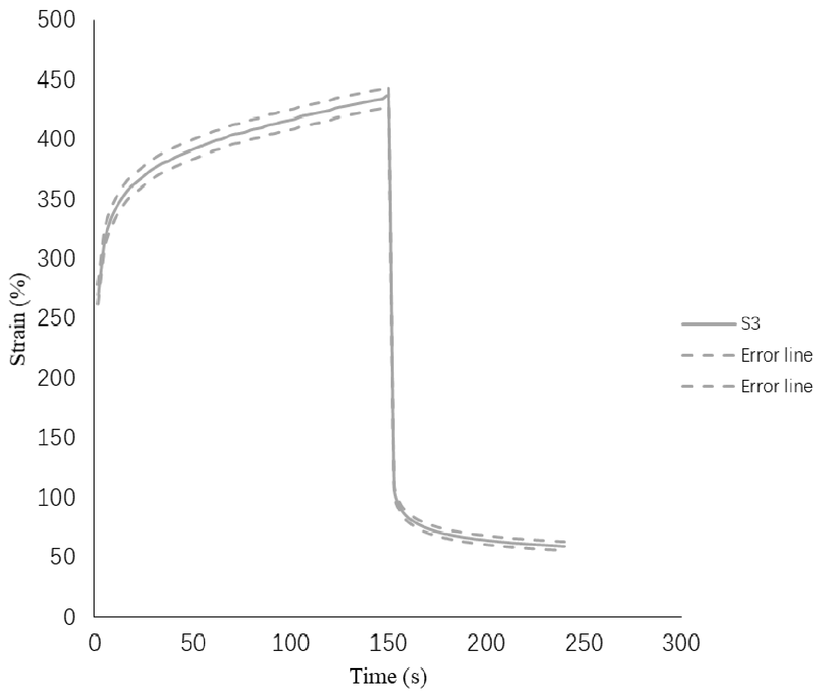

Figure S18. Strain-time plots at constant stress level applied to the sample S3 tested with error line ($n = 3$).

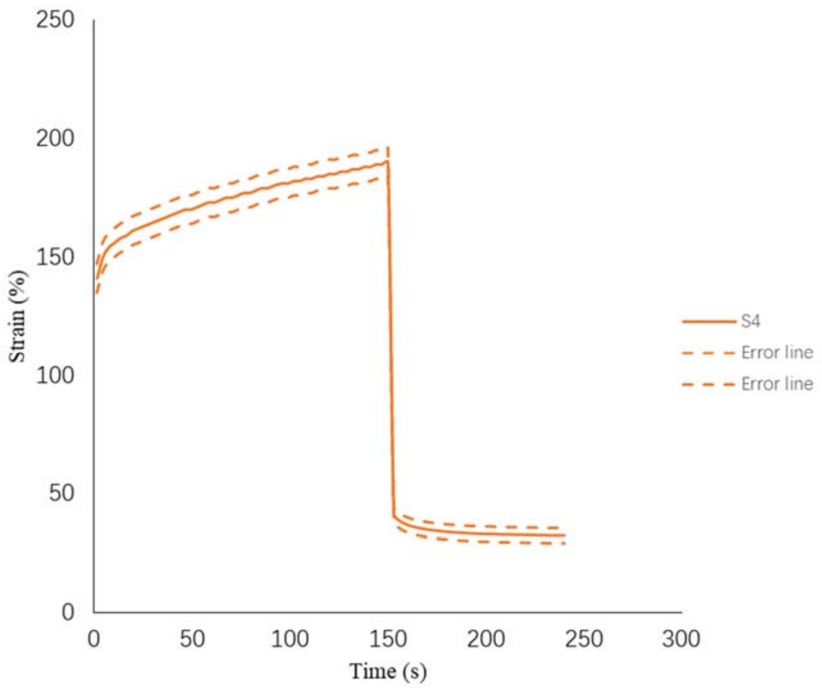

Figure S19. Strain-time plots at constant stress level applied to the sample S4 tested with error line ($n = 3$).

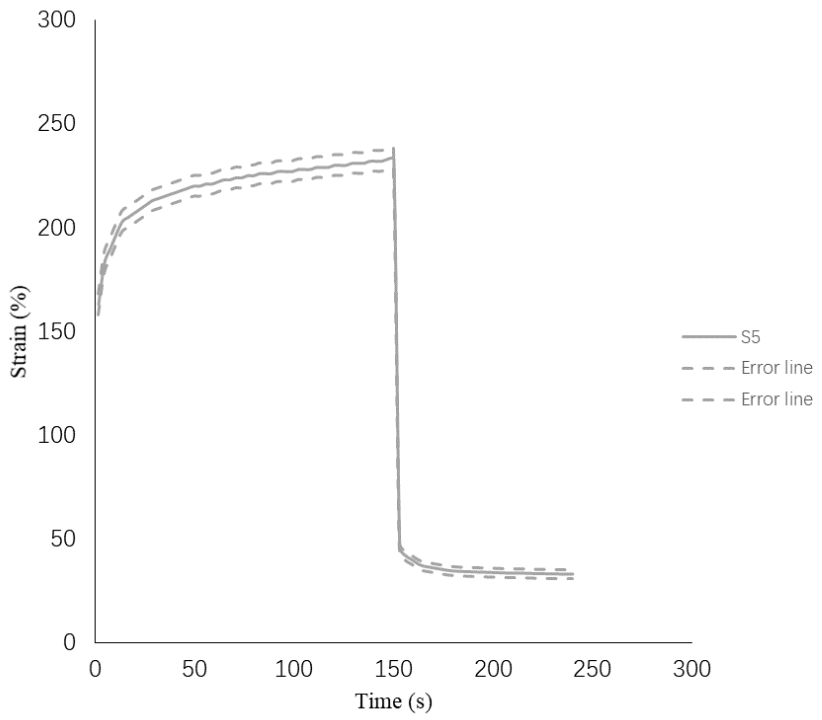

Figure S20. Strain-time plots at constant stress level applied to the sample S5 tested with error line (n = 3).

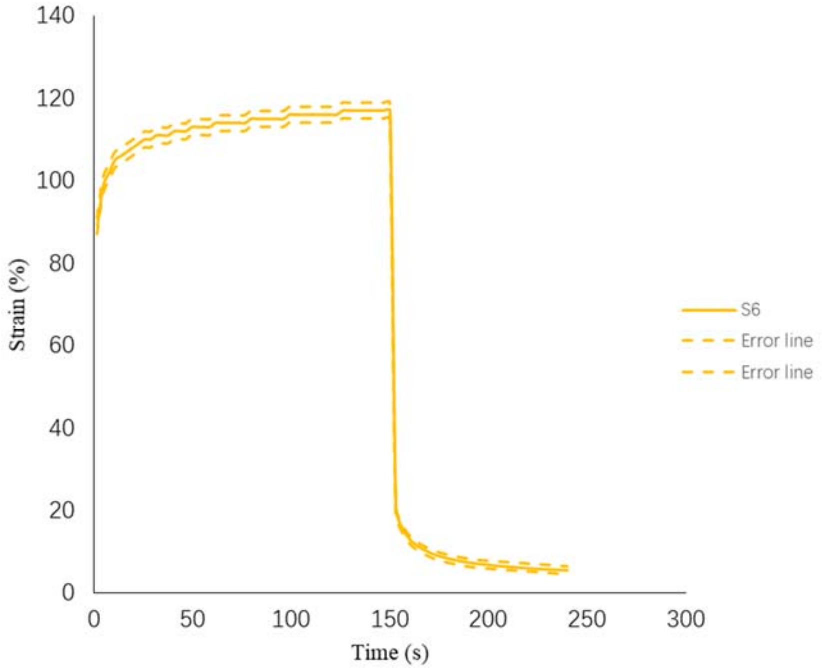

Figure S21. Strain-time plots at constant stress level applied to the sample S6 tested with error line (n = 3).

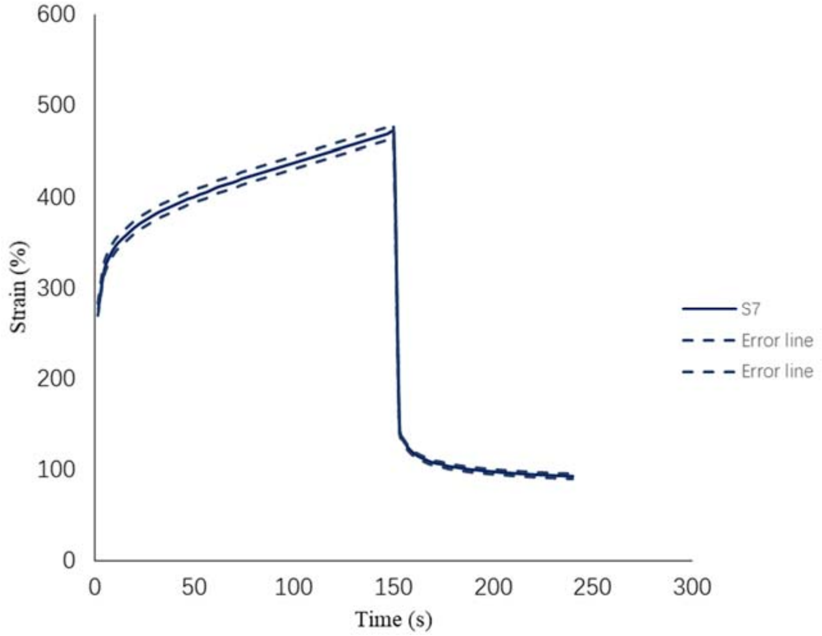

Figure S22. Strain-time plots at constant stress level applied to the sample S7 tested with error line (n = 3).

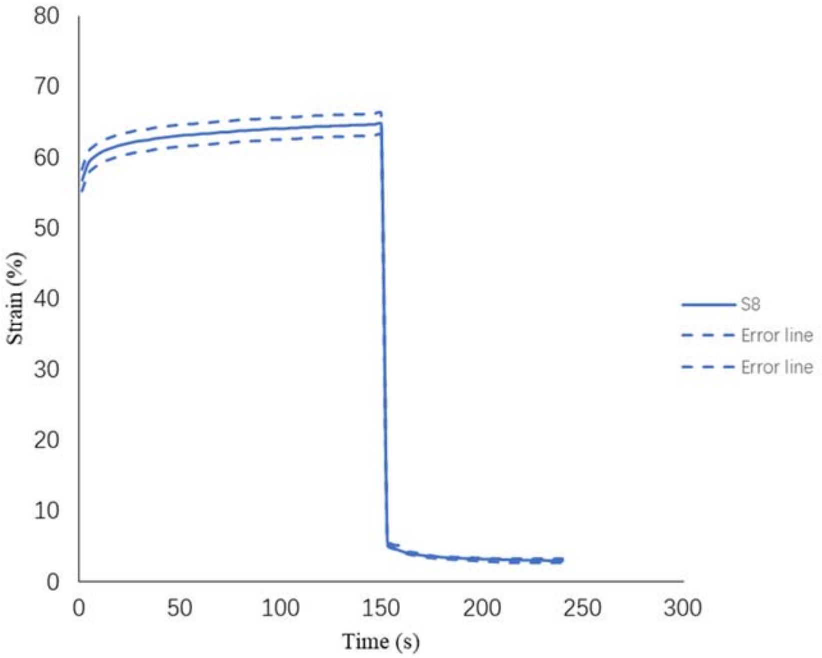

Figure S23. Strain-time plots at constant stress level applied to the sample S8 tested with error line (n = 3).

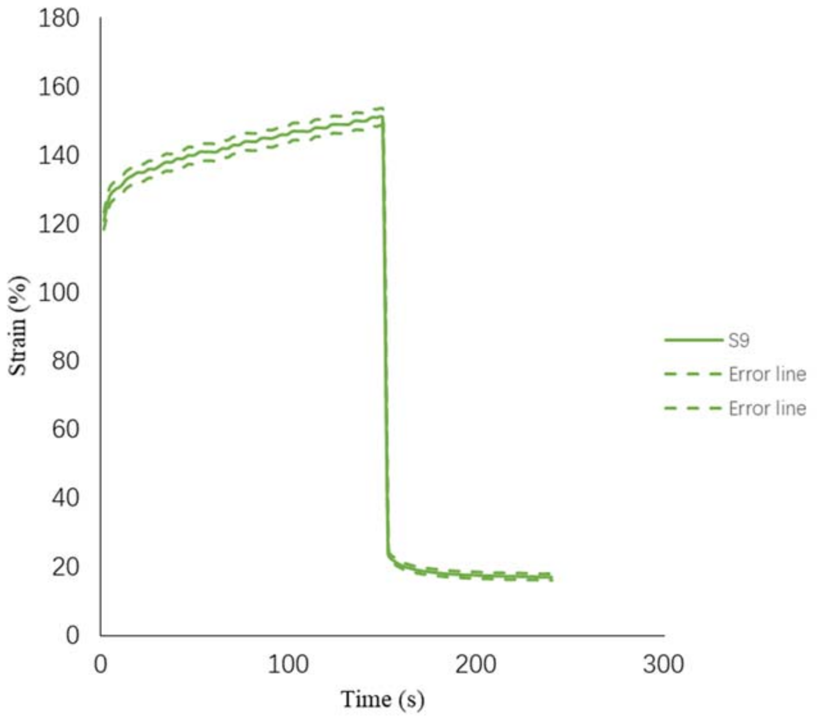

Figure S24. Strain-time plots at constant stress level applied to the sample S9 tested with error line ($n = 3$).

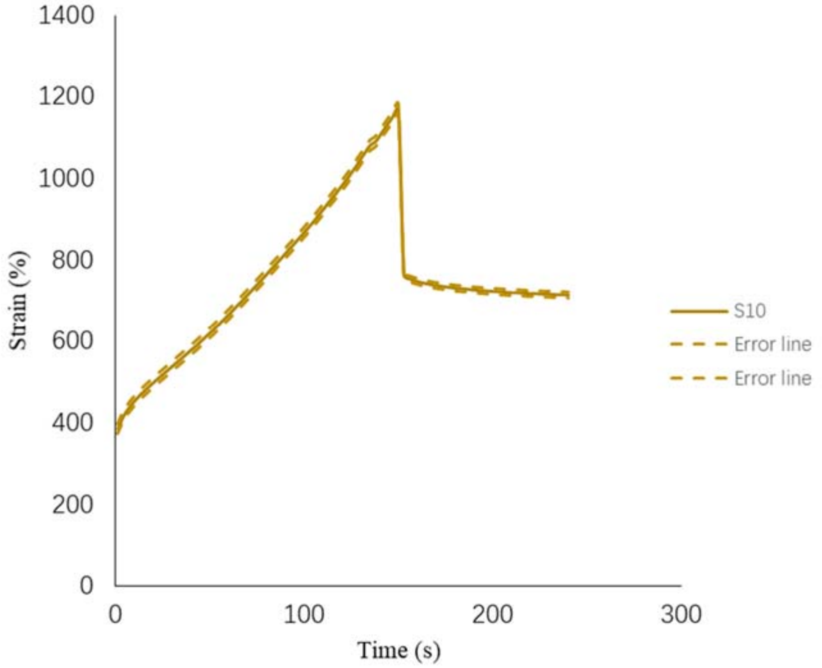

Figure S25. Strain-time plots at constant stress level applied to the sample S10 tested with error line ($n = 3$).

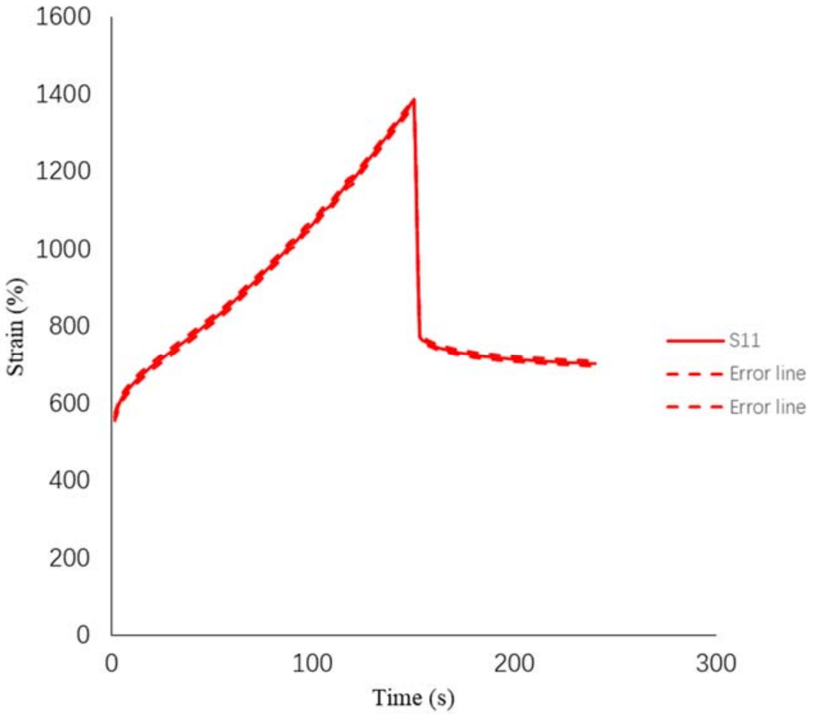

Figure S26. Strain-time plots at constant stress level applied to the sample S11 tested with error line (n = 3).

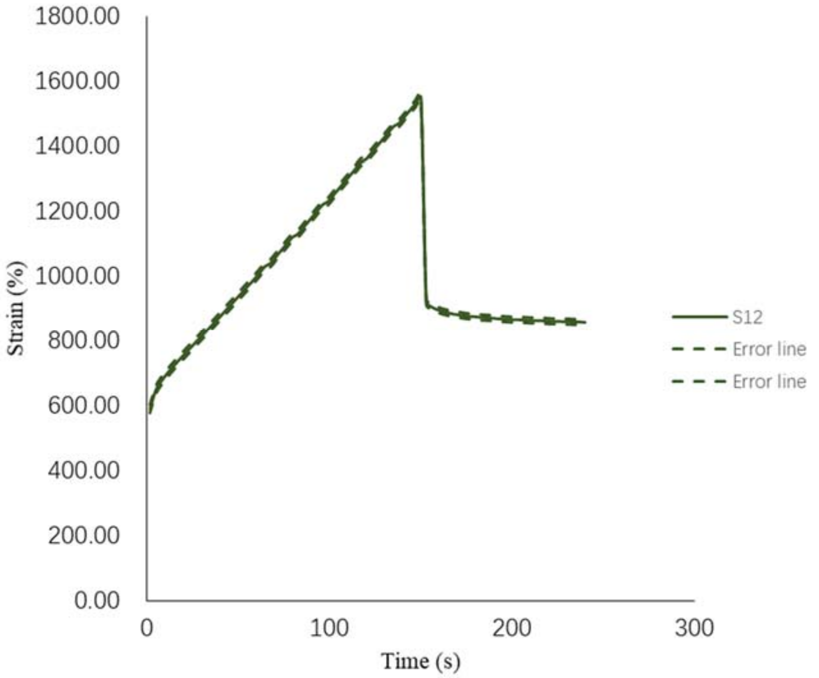

Figure S27. Strain-time plots at constant stress level applied to the sample S12 tested with error line (n = 3).

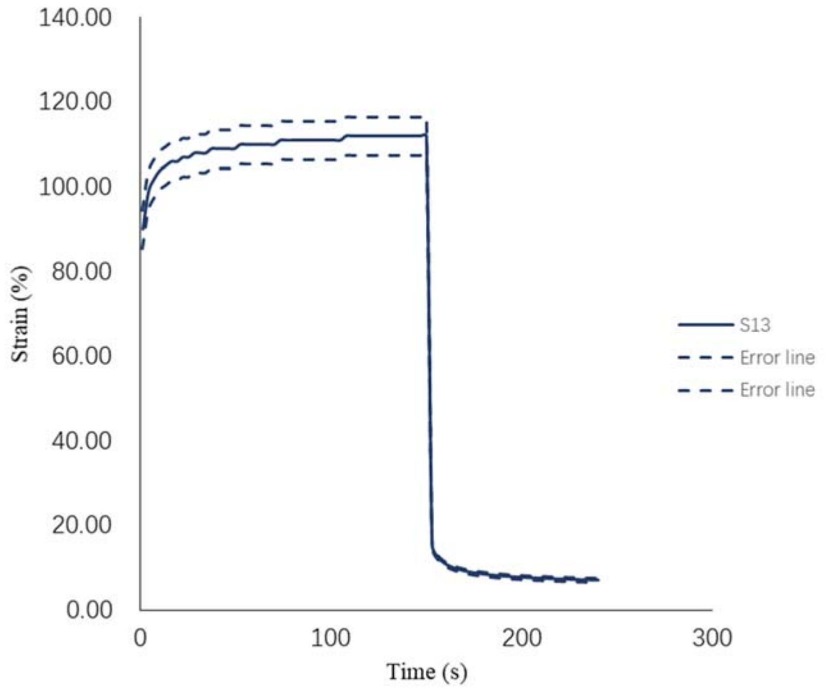

Figure S28. Strain-time plots at constant stress level applied to the sample S13 tested with error line ($n = 3$).

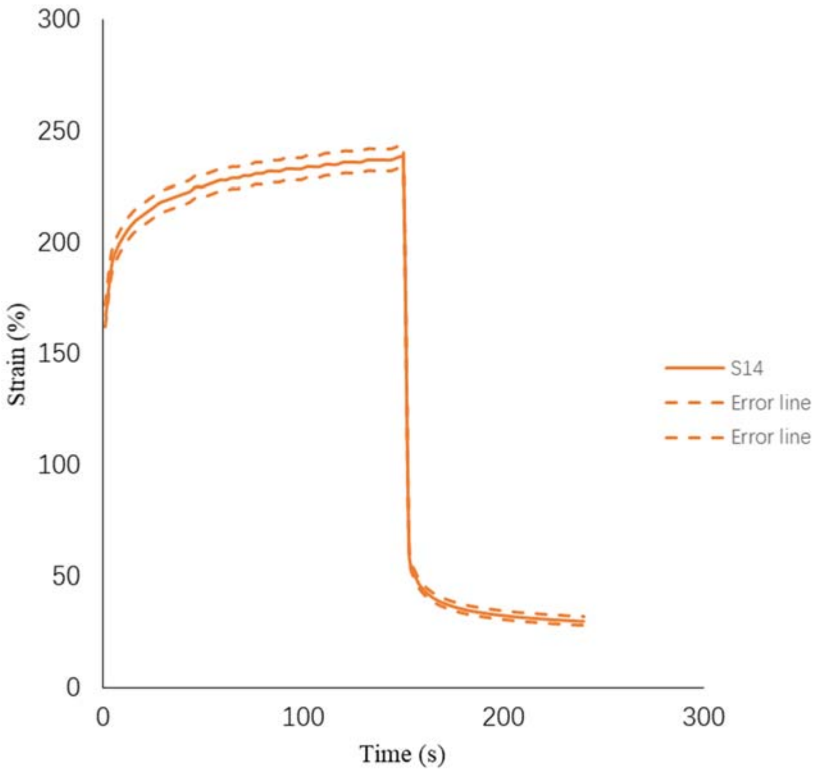

Figure S29. Strain-time plots at constant stress level applied to the sample S14 tested with error line ($n = 3$).

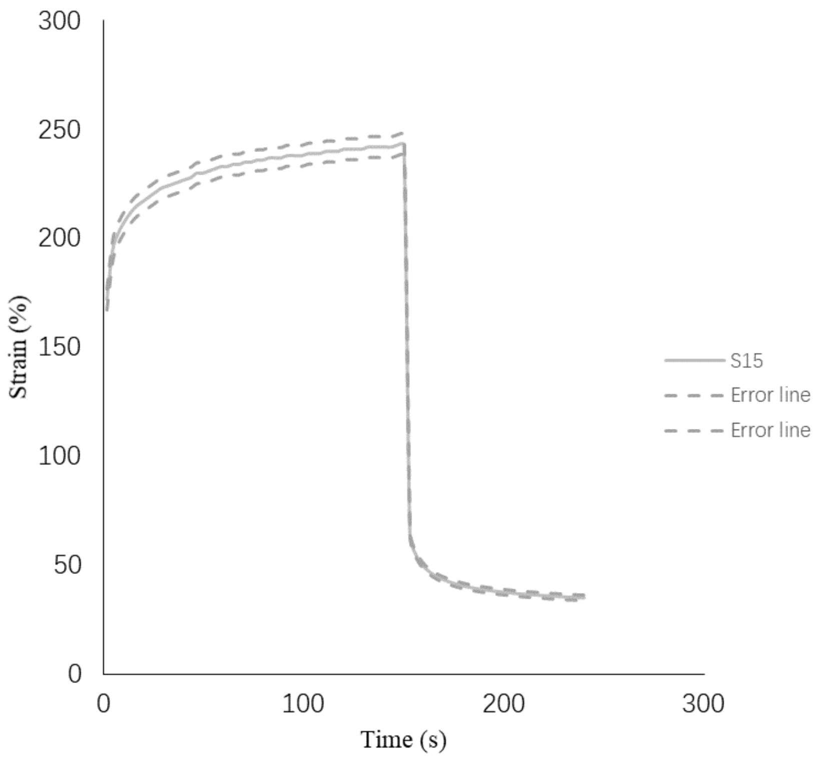

Figure S30. Strain-time plots at constant stress level applied to the sample S15 tested with error line (n = 3).
